# Medipix in space on-board the ISS

**DOI:** 10.1093/jrr/rrt197

**Published:** 2014-03

**Authors:** Lawrence S. Pinsky, J. Idarraga-Munoz, M. Kroupa, H.M. Son, N.N. Stoffle, E.J. Semones, A.A. Bahadori, D. Turecek, S. Pospíšil, J. Jakubek, Z. Vykydal, H. Kitamura, Y. Uchihori

**Affiliations:** 1 Physics Department, University of Houston, 4800 Calhoun Rd, Houston, TX 77201-5005, USA; 2 NASA Johnson Space Center, Houston, TX, USA; 3 Institute for Experimental and Applied Physics, Czech Technical University in Prague, Czech Republic; 4 National Institute for Radiological Sciences, Inage, Japan

## Abstract

On 16 October 2012, five active radiation detectors (referred to by NASA as Radiation Environment Monitors, or REMs) employing the Timepix version of the technology developed by the CERN-based Medipix2 Collaboration were deployed on-board the International Space Station (ISS) using simple USB interfaces to the existing ISS laptops for power, control and readout [ 1– 3]. These devices successfully demonstrated the capabilities of this technology by providing reliable dose and dose-equivalent information based on a track-by-track analysis. Figure 1 shows a sample comparison of the output from all five devices with respect to the on-board tissue equivalent proportional counter (TEPC) for both absorbed dose (top) and dose-equivalent (bottom) as defined in NCRP 142. The lower graph in each set is the TEPC. Several issues were identified and solutions to adjust for them have been included in the analysis. These include items such as the need to identify nuclear interactions in the silicon sensor layer, and to separate penetrating from stopping tracks. The wide effective range in fluence and particle type of this technology was also verified through the highest rates seen during the South Atlantic Anomaly passes and the heavy ions nominally seen in the Galactic Cosmic Rays. Corrections for detector response saturation effects were also successfully implemented as verified by reference to ground-based accelerator data taken at the Heavy-Ion Medical Accelerator Center (HIMAC) facility at the National Institute for Radiological Sciences in Japan, and at the NASA Space Radiation Laboratory (NSRL) at the Brookhaven National Laboratory in New York. Flight hardware has been produced that will be flown on the first launch of the new Orion spacecraft, and flight hardware development is ongoing to accommodate the next generation of this technology as a baseline for radiation monitoring and dosimetry on future operational manned missions.

**Fig 1.**
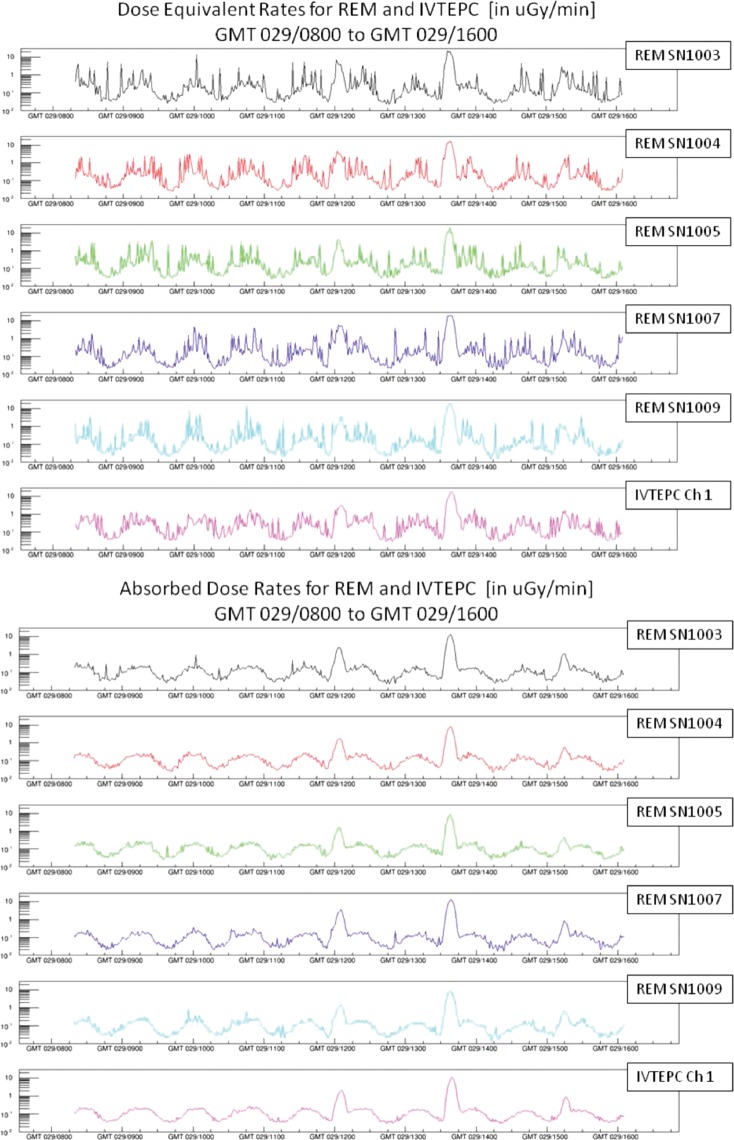
Five ISS REM units compared with ISS IVTEPC in absorbed dose (a) and dose-equivalent (b).

Five ISS REM units compared with ISS IVTEPC in absorbed dose (a) and dose-equivalent (b).
